# Chronic vitamin D deficiency induces lung fibrosis through activation of the renin-angiotensin system

**DOI:** 10.1038/s41598-017-03474-6

**Published:** 2017-06-12

**Authors:** Yongyan Shi, Tianjing Liu, Li Yao, Yujiao Xing, Xinyi Zhao, Jianhua Fu, Xindong Xue

**Affiliations:** 1 Department of Pediatrics, Shengjing Hospital of China Medical University, NO. 36 Sanhao Street, Shenyang, Liaoning 110004 P.R. China; 2 Department of Pediatric Orthopedics, Shengjing Hospital of China Medical University, NO. 36 Sanhao Street, Shenyang, Liaoning 110004 P.R. China

## Abstract

Pulmonary fibrosis, which influences lung function and exacerbates a patient’s condition, is the ultimate stage of many lung diseases. Vitamin D deficiency is associated with pulmonary fibrosis and impaired lung function, but the underlying mechanism has not yet been fully elucidated. Moreover, vitamin D deficiency may cause over-activation of the renin-angiotensin system (RAS), which aggravates extracellular matrix (ECM) deposition and lung fibrosis. This study aims to investigate the effect of chronic vitamin D deficiency on lung fibrosis in otherwise healthy mice and to explore the role of RAS in this process. Mice were depleted of vitamin D through diet control and were compared with healthy subjects. Chronic vitamin D deficiency destructs lung structures, impairs lung development and stimulates ECM deposition. RAS components are also found to increase. These effects seem to worsen with prolonged vitamin D deficiency. By giving RAS blockers, these changes can be largely rescued. However, a smooth muscle relaxant whose regulatory effect on blood pressure is independent of RAS does not show similar effects. This study demonstrated that chronic vitamin D deficiency may induce RAS activation, which subsequently stimulates the expression of profibrotic factors and activates the fibrotic cascade. This profibrotic effect of RAS is independent of elevated blood pressure.

## Introduction

Lung fibrosis is the final stage of many pulmonary diseases. It results from the loss of balance between the production and absorption of extracellular matrix (ECM). Over-activation of fibrotic repair due to lung injury causes thickening of the alveolar wall and collapse of the alveoli. The pathological changes include irreversible pulmonary epithelial cell injury and proliferation and the accumulation of fibroblasts and myofibroblasts as well as collagen deposition. These effects worsen lung function, partly due to the higher resistance to airflow and the decreased capacity to clear pathogens caused by decreased airway diameter^1^.

Vitamin D deficiency (VDD) is closely associated with lung diseases, including asthma, cystic fibrosis, interstitial lung disease, chronic obstructive pulmonary disease (COPD) and respiratory infections^2^. Vitamin D treatment could prevent bleomycin-induced pulmonary fibrosis by delaying or suppressing ultrastructural changes, as well as by attenuating hydroxyproline accumulation and inhibiting myofibroblastic proliferation^3^. Vitamin D intake decreased COPD exacerbation and improved forced expiratory volume in one second (FEV1) in patients with severe and very severe COPD^4^. Vitamin D and its receptor play a vital role in fibrosis of the kidney^5^, peritoneum^6^, liver^7^ and lung^3^. Among these, renal fibrosis is the most studied. Vitamin D plays its antifibrotic role through a negative regulation of the renin-angiotensin system (RAS) and the inhibition of nuclear factor kappa B (NF-κB) and wnt/β-catenin^8^.

Previous studies have suggested a high prevalence of VDD (25(OH)D < 30 nmol/L) and have found an association between vitamin D status and acute respiratory morbidity in preterm infants after birth^9^. VDD is common in people who develop acute respiratory distress syndrome (ARDS). This deficiency of vitamin D appears to contribute to the development of the condition, and approaches to correct VDD in patients at risk of ARDS should be developed^10^. There is a high prevalence of VDD in patients with interstitial lung disease, and it is associated with reduced lung function^11^. Low 25(OH)D levels are associated with pulmonary exacerbations in cystic fibrosis patients^12^. Previously, studies mainly investigated the curative effect of vitamin D analogues or the aggravating effect of VDD on disease models in animals. The influence of VDD on otherwise healthy subjects has received less attention.

VDD promotes the renin-angiotensin system (RAS)^13^, while chronic RAS activation can decrease lung function and compliance by inducing fibrosis^14^. According to these findings, VDD may cause lung fibrosis by activating RAS. However, due to the complex physiological functions of RAS, it is hard to demonstrate whether this is achieved by the direct local effect of RAS or by its classical function of inducing hypertension and subsequent angiosclerosis.

In this study, we prepared a whole-life vitamin D deficient model in mice through dietary depletion. We aimed at exploring: 1. The profibrotic effect of VDD on otherwise healthy subjects; 2. The role of RAS in this process; and 3. Whether the effect of RAS is caused by direct influence or by a regulatory effect on blood pressure.

## Results

A vitamin D deficient diet successfully induced a vitamin D deficiency in the experimental group. After two months of the vitamin D deficient diet, the serum 25(OH)D level was 14.45 ± 1.68 μmol/L in the experimental group compared to a level of 51.16 ± 4.15 μmol/L in the control group (*P* < 0.001). There was no difference in the serum calcium and phosphate levels or somatic growth between the two groups (*P* > 0.05) (Table 1).

**Table 1:** General data of the control and vitamin D deficiency mice.

	Ctrl 2 m	VDD 2 m	*P* value
25(OH)D (μmol/L)	51.2 ± 4.15	14.5 ± 1.68	< 0.001
Calcium (mg/dL)	9.2 ± 0.38	9.1 ± 0.41	> 0.05
Phosphorus (mg/dL)	9.3 ± 0.39	9.4 ± 0.29	> 0.05
Body weight (g)	20.7 ± 0.49	20.5 ± 0.43	> 0.05

Ctrl: control; VDD: vitamin D deficiency.

### Chronic vitamin D deficiency led to structural damage and increased lung fibrosis

**Figure 1:**
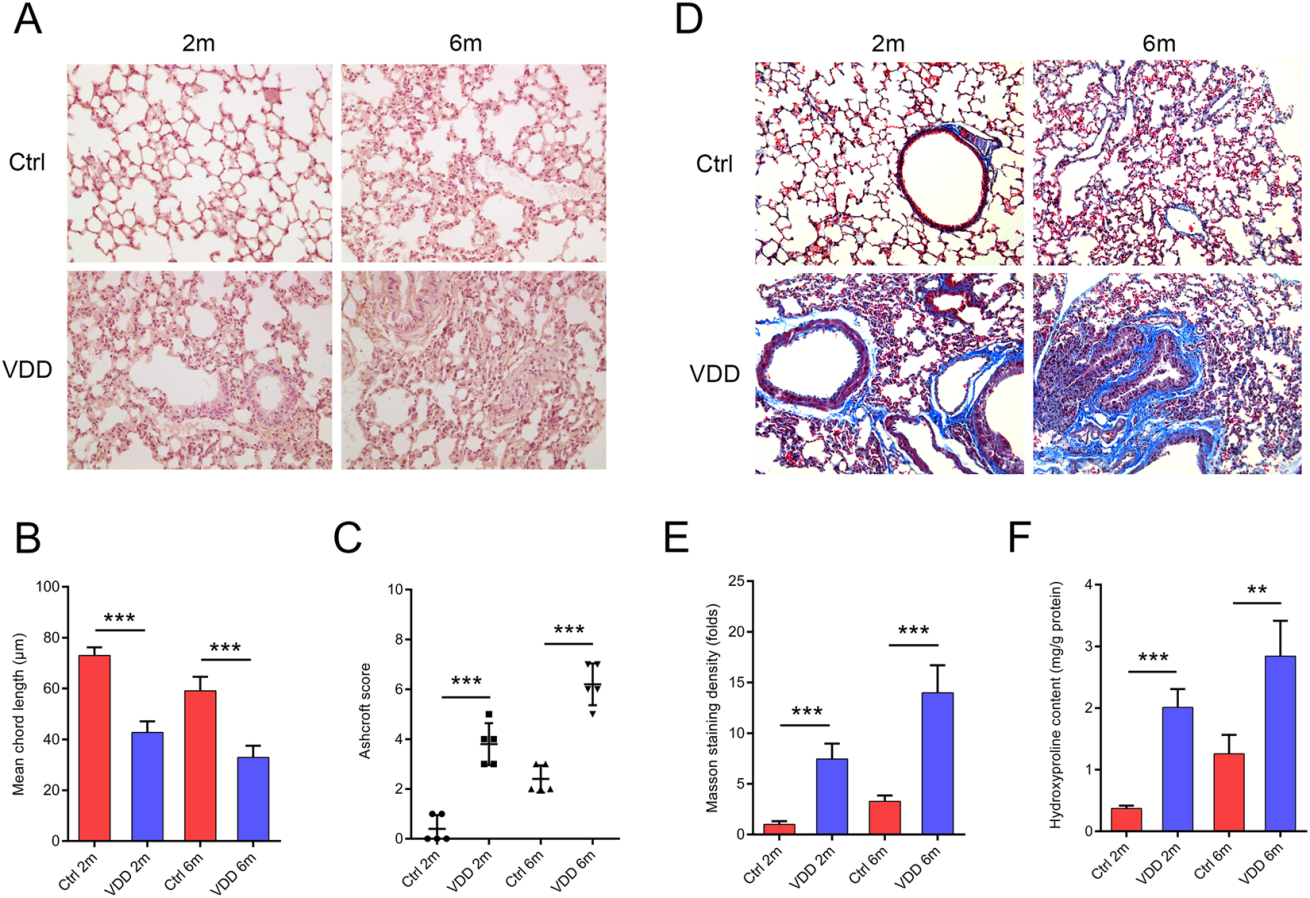
Chronic vitamin D deficiency results in structural damage and increased lung fibrosis, which aggravates with time. (**A**) Haematoxylin and eosin staining of the lung tissue after two months and six months of vitamin D deficiency. Original magnification: 200×. (**B**) Mean chord length of the lungs. ***P <  0.001 Ctrl versus VDD group of the same age (n = 10 in each group). Significant difference existed between Ctrl 2 m group and Ctrl 6 m group (*P* < 0.001). (**C**) Microscopic scoring of the lung tissue according to Ashcroft. ***P < 0.001 Ctrl versus VDD group of the same age (n = 5 in each group). Significant difference existed between Ctrl 2 m group and Ctrl 6 m group (*P* < 0.001). (**D**,**E**) Masson staining and the quantification of collagen density. ***P < 0.001 Ctrl versus VDD group of the same age (n = 5–6 in each group). Significant difference existed between Ctrl 2 m group and Ctrl 6 m group (*P* < 0.001). (**F**) Hydroxyproline content in each group. **P < 0.01, ***P < 0.001 Ctrl versus VDD group of the same age (n = 5–6 in each group). Significant difference existed between Ctrl 2 m group and Ctrl 6 m group (*P* < 0.01). Ctrl: control; VDD: chronic vitamin D deficiency; 2 m: 2 months; 6 m: 6 months. Statistics: Fig. 1B, (**E**,**F**): Independent-sample two-tailed *t*-test; Fig. 1C: Wilcoxon signed rank test.

Chronic VDD resulted in disrupted alveolar structure, underdeveloped lungs and increased Ashcroft scores, which aggravated with time (Fig. 1A). In the vitamin D deficient mice, the alveoli were irregular, distorted or collapsed. The mean chord length, which reflects the alveolar size, was substantially reduced in the vitamin D deficient mice (Fig. 1B). Ashcroft scoring reflected obvious fibrotic changes, especially in the lungs after six months of VDD (Fig. 1C). Masson trichrome staining showed increased collagen deposition (Fig. 1D,E). Increased hydroxyproline, which indicates increased collagen formation, was also noted in the vitamin D deficient mice (Fig. 1F). All of these changes were aggravated with prolonged VDD.

**Figure 2:**
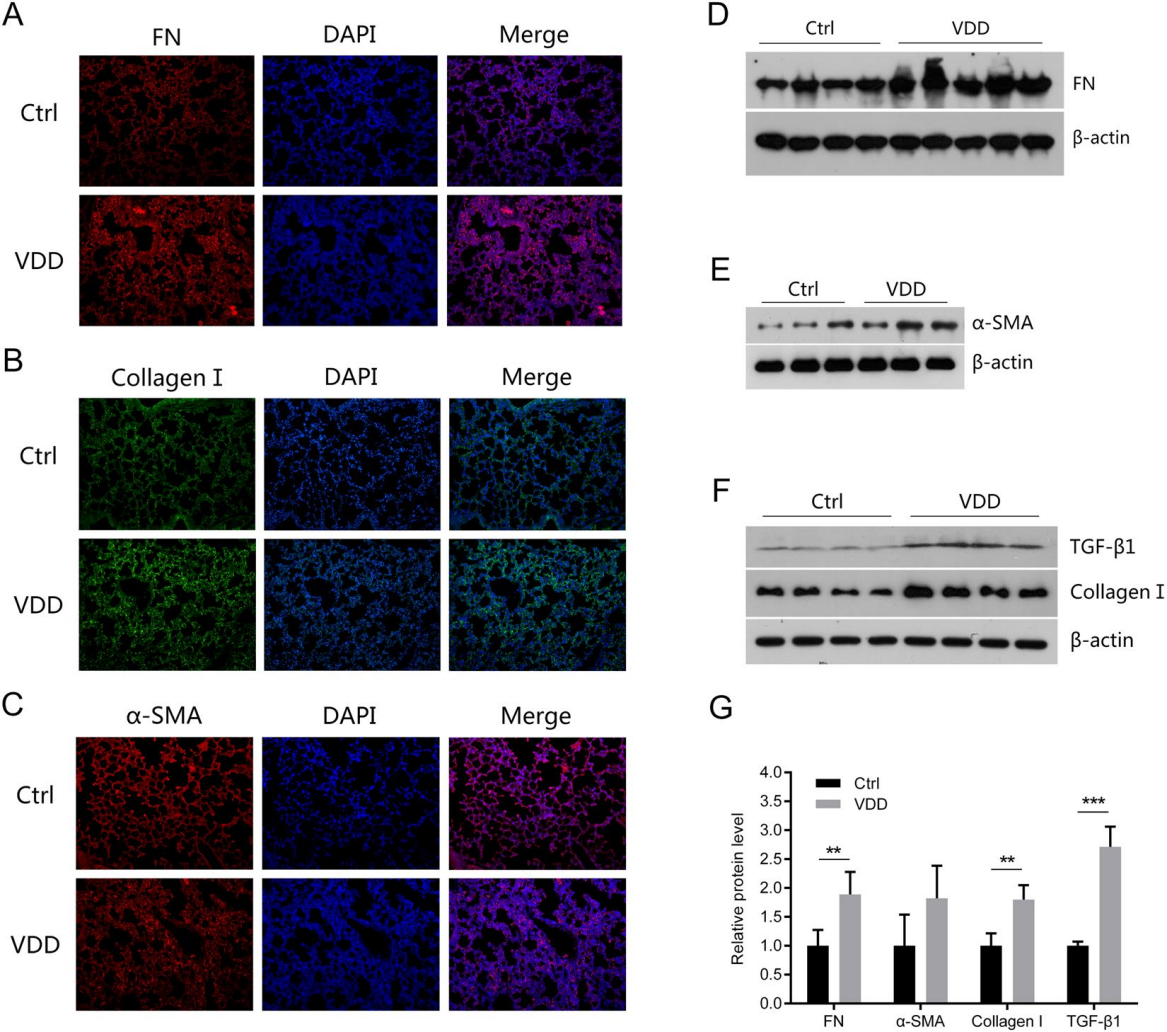
Chronic vitamin D deficiency promotes ECM deposition in the lung. (**A**,**B**,**C**) Immunofluorescence staining of the lung tissue with fibronectin, collagen I and α-SMA after two months of vitamin D deficiency. Original magnification: 200×. (**D**,**E**,**F**) Western blotting of fibronectin, α-SMA, collagen I and TGF-β1. (**G**) Relative expression of the proteins above. **P < 0.01, ***P < 0.001 Ctrl versus VDD group (n = 5–6 in each group). FN: fibronectin; α-SMA: α-smooth muscle actin; TGF-β1: transforming growth factor-β1; Ctrl: control; VDD: chronic vitamin D deficiency. Statistics: Independent-sample two-tailed *t*-test. All electrophoresis was performed under the same conditions, and the gel images were cropped for concise presentation. The uncut images are provided in the Supplementary Materials.

We went on to investigate the expression of fibrosis-related proteins after two months of exposure to VDD. Extracellular matrix proteins, including fibronectin, collagen I and α-smooth muscle actin (α-SMA), together with the pro-fibrotic factor transforming growth factor-β1 (TGF-β1) were evaluated. Immunofluorescence showed a large amount of extracellular matrix deposition, especially in the alveolar walls, resulting in thickening of the alveolar septa (Fig. 2A–C). As shown by Western blotting, the expression levels of the extracellular matrix proteins in the vitamin D deficient group were twice as high as those in the control group. The expression of TGF-β1 increased even more (Fig. 2D–F). Quantification demonstrated a significant difference in the expression of all the proteins except for α-SMA (Fig. 2G).

### Vitamin D deficiency activated pulmonary RAS expression

**Figure 3:**
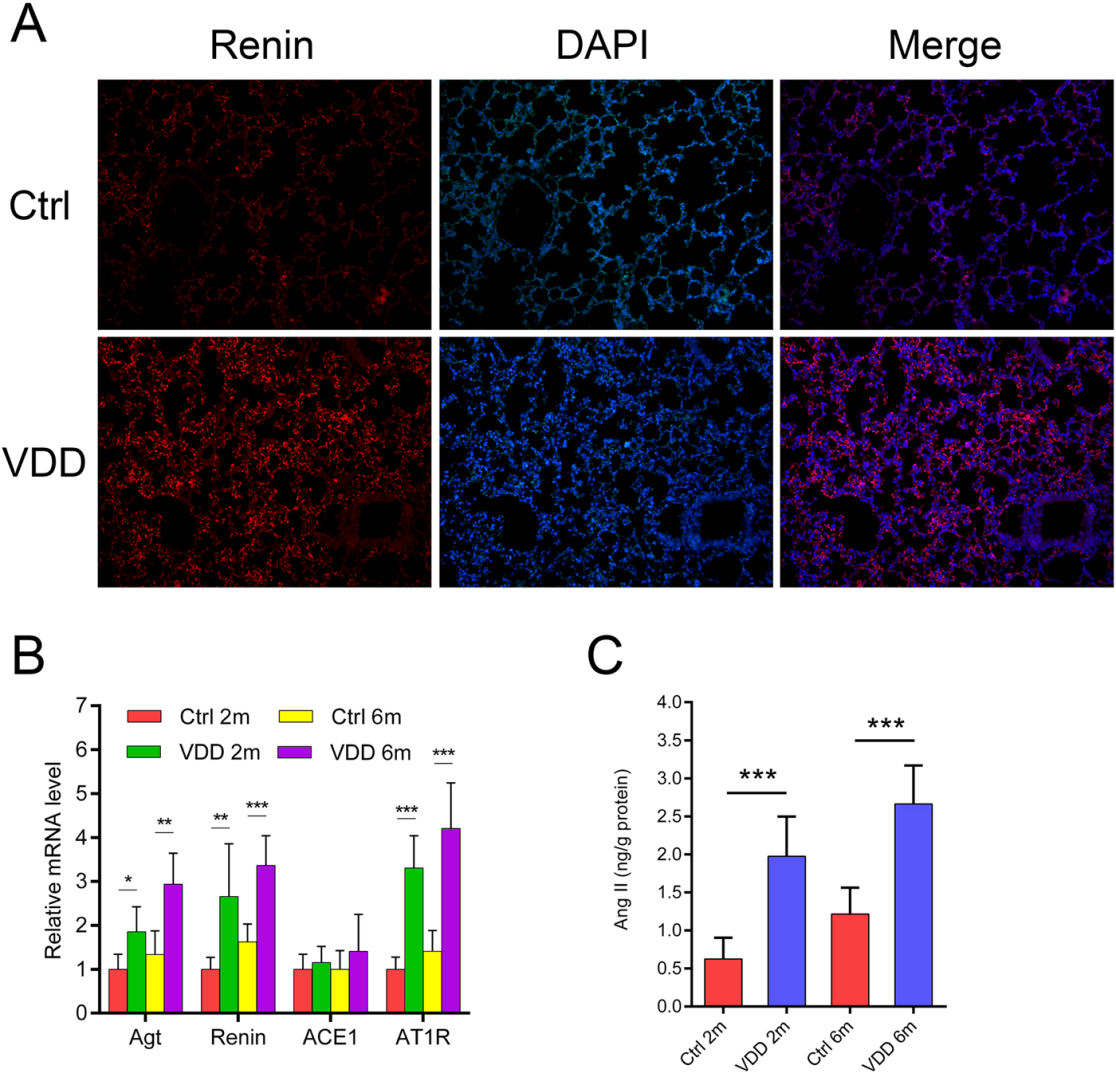
Vitamin D deficiency activates pulmonary RAS. (**A**) Immunofluorescence staining of the lung tissue with renin after two months of vitamin D deficiency. Original magnification: 200×. (**B**) Relative mRNA expressions of RAS components by real-time PCR. *P < 0.05, **P < 0.01, ***P < 0.001 Ctrl versus VDD group of the same age (n = 5–6 in each group). (**C**) Serum angiotensin II concentration in the mice after 2 months and six months of vitamin D deficiency. ***P < 0.001 Ctrl versus VDD group of the same age (n = 5–6 in each group). Agt: angiotensinogen; Ang: angiotensin; ACE1: angiotensin converting enzyme 1; AT1R: angiotensin type 1 receptor; Ctrl: control; VDD: chronic vitamin D deficiency; 2 m: 2 months; 6 m: 6 months. Statistics: Independent-sample two-tailed *t*-test.

Since VDD was highly related to RAS activation, we examined the expression of renin by immunofluorescence. Renin was significantly up-regulated by chronic VDD (Fig. 3A). Then, we tested other components of RAS. Real-time PCR demonstrated an elevation in angiotensinogen (Agt), renin, angiotensin converting enzyme 1 (ACE1) and angiotensin type 1 receptor (AT1R) levels in the lungs of the vitamin D deficient mice. With prolonged VDD, the difference became larger (Fig. 3B). The serum level of angiotensin (Ang) II was also found to increase with time, indicating more angiotensin II in circulation (Fig. 3C).

### Vitamin D deficiency induced lung fibrosis through RAS activation independent of high blood pressure

VDD causes RAS activation and blood pressure elevation, both of which might result in lung fibrosis. To further test the relationship between RAS activation and lung fibrosis, we gave RAS blockers, including the renin blocker aliskiren and the AT1R blocker losartan, to the vitamin D deficient mice and observed their effect on lung fibrosis. We also included a smooth muscle relaxant, the hydralazine group, in which the blood pressure was normalized independent of RAS, to test whether the anti-fibrotic effect of the RAS blockers was due to their role in blood pressure control. The effect of losartan, aliskiren and hydralazine on RAS components was tested in preliminary experiments (see Supplementary Figure 1).

**Figure 4:**
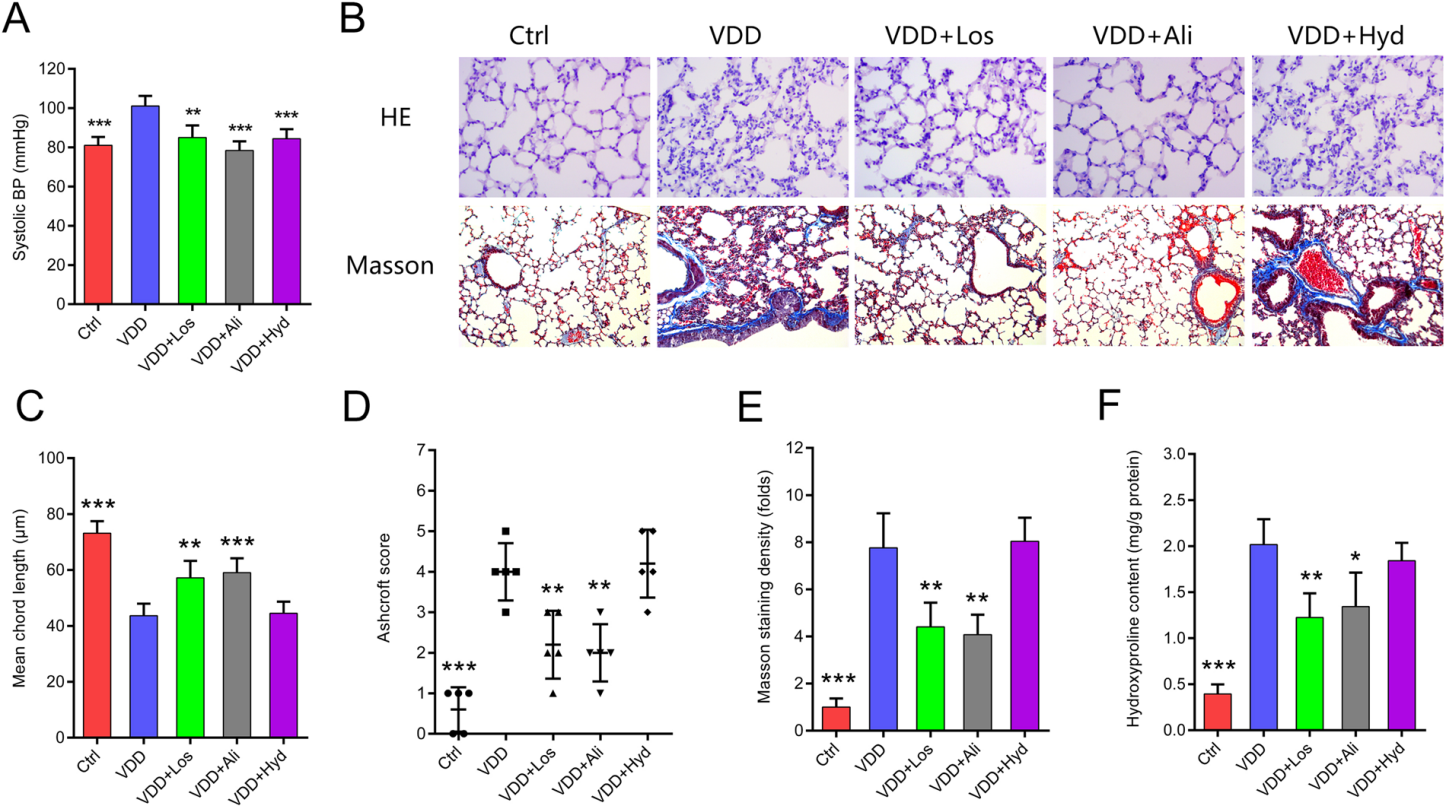
RAS blockers losartan and aliskiren rescue lung structure damage induced by chronic vitamin D deficiency, but the vascular relaxant hydralazine does not have this effect. (**A**) Systolic blood pressure in the control and experimental groups. **P < 0.01, ***P < 0.001 compared to the VDD group (n = 5–6 in each group). (**B**) H&E and Masson staining of the lung tissue from each group. Original magnification: 200×. (**C**) Mean chord length of the lungs for each group. **P < 0.01, ***P < 0.001 compared to the VDD group (n = 5–6 in each group). (**D**) Microscopic scoring of the lung tissue according to the Ashcroft scoring system in each group. **P < 0.01, ***P < 0.001 compared to the VDD group (n = 5–6 in each group). (**E**) Quantification of the Masson staining density in each group. **P < 0.01, ***P < 0.001 compared to the VDD group (n = 5–6 in each group). (**F**) Tissue hydroxyproline content in each group. *P < 0.05, **P < 0.01, ***P < 0.001 compared to the VDD group (n = 5–6 in each group). BP: blood pressure; Ctrl: control; VDD: chronic vitamin D deficiency; VDD + Los: chronic vitamin D deficiency treated with losartan; VDD + Ali: chronic vitamin D deficiency treated with aliskiren; VDD + Hyd: chronic vitamin D deficiency treated with hydralazine. Statistics: Two-way ANOVA and Dunnett *t*-test.

The dissolvent PBS did not bring any change to the lung structure (see Supplementary Figure 2). All the three drugs brought the blood pressure back to normal or nearly normal (Fig. 4A). Aliskiren and losartan restored the collapsed alveoli, increased the chord length and reduced the extracellular matrix deposition surrounding the cells, blood vessels and bronchia. The Ashcroft score was significantly improved and the hydroxyproline concentration in lung lysates decreased. However, hydralazine did not show such a protective effect (Fig. 4B–E).

**Figure 5:**
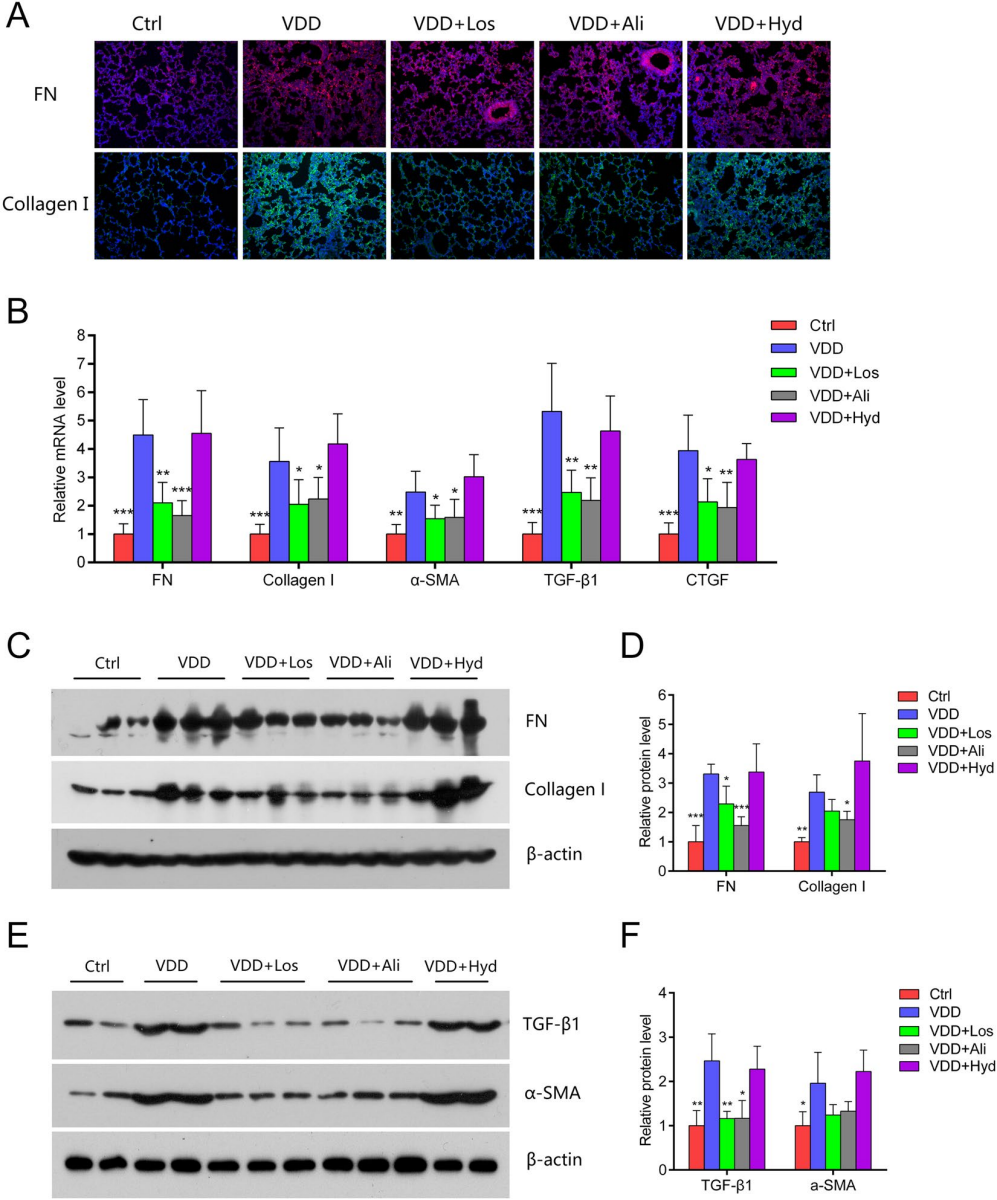
RAS blockers losartan and aliskiren reduce lung ECM deposition, while the vascular relaxant hydralazine does not have this effect. (**A**) Immunofluorescence staining of the lung tissue with fibronectin and collagen I in each group. Original magnification: 200×. (**B**) Relative mRNA expressions of fibronectin, collagen I, α-SMA, TGF-β1 and CTGF by real-time PCR. *P < 0.05, **P < 0.01, ***P < 0.001 compared to the VDD group (n = 5–6 in each group). (**C**,**D**) Western blotting and protein quantification of fibronectin and collagen I. *P < 0.05, **P < 0.01, ***P < 0.001 compared to the VDD group (n = 5–6 in each group). (**E**,**F**) Western blotting and protein quantification of TGF-β1 and α-SMA. *P < 0.05, **P < 0.01 compared to the VDD group (n = 5–6 in each group). FN: fibronectin; α-SMA: α-smooth muscle actin; TGF-β1: transforming growth factor-β1; CTGF: connective tissue growth factor; Ctrl: control; VDD: chronic vitamin D deficiency; VDD+Los: chronic vitamin D deficiency treated with losartan; VDD+Ali: chronic vitamin D deficiency treated with aliskiren; VDD+Hyd: chronic vitamin D deficiency treated with hydralazine. Statistics: Two-way ANOVA and Dunnett *t*-test. All electrophoresis was performed under the same conditions, and the gel images were cropped for concise presentation. The uncut images are provided in the Supplementary Materials.

Immunofluorescence showed that aliskiren and losartan reduced ECM deposition and down-regulated the mRNA expressions of ECM components and pro-fibrotic factors. Similarly, hydralazine did not show any influence on these factors (Fig. 5A). Western blotting and real-time PCR showed the corresponding changes in fibrotic marker proteins (Fig. 5B–F).

## Discussion

This study demonstrated that chronic VDD resulted in lung fibrosis, partly through the activation of the renin-angiotensin system. The AT1 blocker losartan and the renin blocker Aliskiren could alleviate lung fibrosis induced by chronic VDD. This effect was independent of the regulatory role of RAS on blood pressure.

Generally there are three types of VDD models: prenatal, postnatal and whole-life VDD. VDD is exclusively achieved through diet and light control, as was done in our study. By using a whole-life VDD model, one study found that VDD causes deficits in lung function that cannot be explained by somatic growth and lung volume^1^. A later study on pre- and postnatal VDD found that prenatal VDD caused diminished tracheal diameter, tracheal cartilage thickness and alveolar simplification which led to increased airway resistance and decreased pulmonary compliance, while postnatal VDD had little influence on tracheal morphology and lung function^15^. Postnatal vitamin D supplementation could improve lung function but not normalize tracheal abnormalities. Another similar study found that the increase in inflammatory molecules, airway smooth muscle mass and baseline airway resistance were caused largely by VDD *in utero* and postnatal supplementation can do nothing but relieve airway hyperresponsiveness^16^. The changes, however, mainly happen in the latter stage of alveolar development because comparisons between VDD and normal subjects did not show significant difference in protein expressions at E14.5 and E17.5^17^.

Previous studies on VDD exclusively focused on its aggravating effect on pre-existing lung diseases that subsequently led to lung fibrosis. Those diseases are mostly chronic, including bronchopulmonary dysplasia, asthma, cystic fibrosis, COPD and interstitial lung disease^18, 19^. Bronchopulmonary dysplasia attacks premature births and proceeds into childhood. Asthma generally starts in childhood or early adulthood, while others attack mostly adults and the aged. This study, however, aimed to explore the pro-fibrotic role of VDD itself. Since these diseases cover almost the entire span from in uterus to old age and fibrosis may happen any time in this span, we used a whole-life VDD model to stay in synchronism with the process of the diseases.

The role of vitamin D in tissue fibrosis has been demonstrated in many organs. Vitamin D analogues were curative in fibrosis models of the kidney^5^, peritoneum^6^, liver^7^ and lung^3^. VDD worsened 2,4,6-trinitrobenzene sulfonic acid-induced intestinal fibrosis in mice^20^. Serum TGF-β levels were inversely correlated with the vitamin D levels in patients with bone marrow fibrosis^21^. VDD has been especially associated with lung fibrosis. It has been shown to increase pulmonary exacerbations and decrease lung function in patients with cystic fibrosis^12^. However, the question of whether vitamin D could aggravate pre-existing fibrosis or induce fibrosis itself remains unclear. In chronic vitamin D deficient mice, alveolar simplification and stunted lung development were present right after birth (see Supplementary Figure 3). Two months after birth we observed pulmonary fibrosis, the accumulation of collagen and the disruption of normal structures. Increased collagen secretion causes stiffness of the tissue and a loss of elasticity. Uncontrolled myofibroblast proliferation, represented by increased α-SMA levels, adds to the deposition of ECM. In this way, the alveolar structure is destroyed, leading to a reduced mean free distance of the air space. These results showed that chronic VDD could lead to lung fibrosis. This pathological change would aggravate with prolonged VDD.

Vitamin D and RAS are so closely related that hypovitaminosis D has been described as the other face of RAS activation^22^. Vitamin D receptor (VDR) knockout mice showed an over-expression of renin, which transformed more angiotensinogen into angiotensin II, so that the animals would increase water intake and intestinal salt absorption^13^. Vitamin D could reduce the abnormal increase in RAS in pancreatic islets^23^. By activating VDR, vitamin D reduces apoptosis through a blockade of RAS in morphine-induced T cell apoptosis^24^. Chronic RAS activation has been shown to promote lung fibrosis, causing an increased expression of ECM proteins and fibrogenetic factors as well as decreased pulmonary compliance^14^.

Because the activation of RAS has been reported to induce lung fibrosis both in transgenic animals and in disease models^14, 25, 26^, it was reasonable to hypothesize that the lung fibrosis observed in vitamin D deficient mice is associated with the increased expression of RAS components. To clarify this, we administered RAS antagonists to the vitamin D deficient mice. Losartan is a blocker of AT1R, which prohibits angiotensin II from binding to AT1R.Therefore the expression of renin is increased due to a negative feedback. Aliskiren blocks the production of renin. Decreased renin up-regulates the expression of angiotensinogen also through a negative feedback. The renin antagonist aliskiren and the AT1R blocker losartan could significantly rescue the tissue damage and alleviate lung fibrosis. This protective effect of RAS blockers has also been reported on lacrimal gland fibrosis in chronic graft-versus-host disease, as well as in bleomycin-induced and radiation-induced lung fibrosis^27–29^.

However, in our study, the influence of RAS may be confounded by its regulatory effect on blood pressure, because hypertension is an independent risk factor of lung fibrosis and may be induced by RAS activation. Thus, we gave hydralazine to the vitamin D deficient mice as a control, which could restore normal blood pressure without interfering with RAS. Hydralazine did not show any preservative effect, indicating that the induction of lung fibrosis by RAS was not due to hypertension.

In chronic vitamin D deficient mice, the expression of renin induces more angiotensinogen to be transformed into angiotensin II, which, by combining with the angiotensin type 1 receptor, activates TGF-β1. TGF-β1 plays a vital role in preserving ECM, inducing fibroblast proliferation and transforming fibroblasts into myofibroblasts^24^. Activated fibroblasts deposit collagen I, an important component of ECM, which causes the thickening of the alveolar walls^30^. Increased numbers of myofibroblasts secrete more α-SMA, as observed in our study. Meanwhile, TGF-β1 stimulates the expression of angiotensinogen and the angiotensin type 1 receptor, forming the angiotensin-TGF-β1 crosstalk^30^. This crosstalk further enhances the profibrotic effect of angiotensin.

Still other mechanisms might explain the role of VDD in lung fibrosis, including the nuclear factor-κB and wnt/β-catenin pathways^8^, and the failure to address to them is one limitation of this study. Moreover, we did not testify our findings in the human population. Large-scale population-based studies are still needed to confirm our conclusions.

In summary, chronic VDD may induce over-activation of RAS, which subsequently stimulates the expression of TGF-β1 and activates the fibrotic cascade. Structural damage and ECM deposition aggravate with prolonged deficiency. This effect of RAS is independent of the classical function of blood pressure regulation. Our study draws attention to chronic VDD in the population, which might be a cause of lung fibrosis.

## Materials and Methods

### Animals

This study was approved by the Institutional Ethical Committee of Shengjing Hospital, China Medical University. All of the procedures were performed in accordance with the approved guidelines. Male and female four-week-old C57BL/6 J mice were provided by the Centre for Experimental Animals of China Medical University. The mice were kept in specific pathogen-free static cages under ultraviolet B-free incandescent light to minimize endogenous vitamin D production. Chow pellets and tap water were available *ad libitum*.

### Vitamin D deficient diet

A vitamin D deficient diet (Harlan Teklad, Madison, WI, USA) was given to female mice from four weeks before mating until the weaning of the pups. The male mice used for mating were given a normal diet (Harlan Teklad, Madison, WI, USA). The offspring of the vitamin D deficient mice were used in this study and were given a vitamin D deficient diet until the end of the experiments. The control group was given a normal diet and was kept in a 12-hour light and dark circle.

### Special treatment

Special treatment with the following drugs was given to selected groups at two month of age. Losartan (Cozar, Merck & Co., Whitehouse Station, NJ, USA) was dissolved in distilled drinking water and given orally to the mice at a dose of 10 mg/kg/day for two weeks before sacrifice^31^. Both aliskiren (Raziles, Novartis, Switzerland) and hydralazine (Sigma, St. Louis, USA) were dissolved in PBS and administered through a daily intraperitoneal injection at the dose of 20 mg/kg/day for two weeks before sacrifice^14^. All of the procedures were approved by the Institutional Ethical Committee of Shengjing Hospital, China Medical University and performed in accordance with the approved guidelines.

### Measurement of serum 25(OH)D levels

Eight-week-old mice were anesthetized by intraperitoneal injection with a cocktail of xylazine (Rompun 2%; Bayer AG, Leverkusen, Germany) and ketamine (Ketavest; 100 mg/ml; Pfizer, Inc., New York, NY, USA). Blood samples were collected from the inferior vena cava. Then, the samples were kept overnight at 4 °C and were centrifuged at 4 °C for 10 minutes at 10, 000×*g*. The serum 25(OH)D levels (nmol/L) were measured using a commercial 25-hydroxyvitamin D RIA kit (Immunodiagnostic Systems PLC, Boldon, Tyne & Wear, UK) according to the manufacturer’s instructions.

### Blood pressure

Systolic ventricular blood pressure was measured and averaged from at least 10 cardiac cycles by a programmable tail-cuff sphygmomanometer (Softron BP-98A, Softron, Tokyo, Japan) as described previously^32^.

### Histology

The lungs were collected and sectioned at the thickness of 4 μm. The slides were stained with H&E (Beyotime Institute of Biotechnology, Haimen, China) at room temperature. Microscopic lung fibrosis scoring was done using the Ashcroft scale^33^. Microscopic lung injury scoring was done using a novel acute lung injury scoring system^34^. The mean score of five fields was recorded as the final score of the mouse. Every group included 10 mice, and two pathologists were blind to the study design.

Masson-Trichrome staining was done using a commercial kit (Beyotime Institute of Biotechnology, Haimen, China) according to the instructions. ECM deposition was analysed using Image-Pro Plus 6.0 software (Media Cybernetics, Rockville, MD, USA). Immunofluorescence was used to locate and evaluate renin expression and ECM deposition. The antibodies and their concentrations were as follows: anti-renin (1:100 dilution; Santa Cruz), anti-fibronectin (1:200 dilution; Abcam), anti-collagen I (1:200 dilution; Abcam) and anti-α-SMA (1:200 dilution; EMD Millipore), and these were subsequently conjugated with Alexa Fluor 555 or 488 secondary antibodies (Invitrogen, Thermo Fisher Scientific, Waltham, MA, USA). The antigens were visualized using a Leica DFC425 fluorescence microscope [Leica Microsystems (Schweiz) AG, Heerbrugg, Switzerland].

### Real-time PCR

Total RNA was isolated from the lung tissues using the TRIzol reagent (Invitrogen). First-strand cDNAs were synthesised with a PrimeScript RT reagent kit (Takara Biotechnology Co., Ltd., Dalian, China). Real-time PCR was performed in the 20 μl volume reaction mixture using an SYBR-Green PCR reagent kit (Clontech Laboratories, Mountain View, CA, USA) and a Bio-RAD IQ5 real-time system. The 2-ΔΔCq formula was used to quantify the relative expression of the mRNA. β-2 microglobulin (B2M) was used as an internal control. The sequences of the PCR primers are provided in Table 2.

**Table 2:** Primer sequences used for the real-time PCR.

Primer name	Forward (5′-3′)	Reverse (3′-5′)
mouse Agt	TTTATCCACTGACCCAGTTC	CTGAGAGAAACCTCTCATCG
mouse Renin	GGACACTGGTTCATCCTTTA	TGTCTCTCCTGTTGGGATAC
mouse ACE1	AGCATCACCAAGGAGAACTA	ACTGGAACTGGATGATGAAG
mouse AT1R	GAAGAACAAGCCAAGAAATG	AATACGCTATGCAGATGGTT
mouse FN	CGAGGTGACAGAGACCACAA	CTGGAGTCAAGCCAGACACA
mouse Collagen I	GCAGGTTCACCTACTCTGTCCT	CTTGCCCCATTCATTTGTCT
mouse α-SMA	GAGGCACCACTGAACCCTAA	CATCTCCAGAGTCCAGCACA
mouse TGF-β1	TGGAGCAACATGTGGAACTCT	CCTGTATTCCGTCTCCTTGGT
mouse CTGF	GTGTGCACTGCCAAAGATGGTG	CAGCTTGACCCTTCTCGGGAA
mouse B2M	CGGCCTGTATGCTATCCAGA	GGGTGAATTCAGTGTGAGCC

Agt: angiotensinogen; ACE1: angiotensin converting enzyme 1; AT1R: angiotensin II type 1 receptor; FN: fibronectin; α-SMA: α-smooth muscle actin; TGF-β1: transforming growth factor-β1; CTGF: connective tissue growth factor; B2M: β-2-microglobulin.

### Western blot

Total protein was extracted from the lung tissue. Equal amounts of proteins (50 μg per lane) were separated by 10% polyacrylamide gel electrophoresis, and the proteins were transferred electrophoretically onto polyvinylidene difluoride membranes (EMD Millipore, Billerica, MA, USA). The membranes were then incubated with primary antibodies. The primary antibodies and their concentrations were as follows: anti-fibronectin (1:2,000 dilution; Abcam), anti-collagen I (1:2,000 dilution; Abcam), anti-α-SMA (1:1,000 dilution; EMD Millipore), and anti-TGF-β1 (1:1,000 dilution; Abcam).

### ELISA assay

Plasma Ang II levels were determined by an AssayMax Angiotensin II ELISA kit (AssayPro, St Charles, MO, USA) according to the instructions.

### Hydroxyproline levels

Newly harvested lung tissue was analysed using the acid hydrolysis method with a commercial kit (Nanjing Jiancheng Bioengineering Institute, China) according to the manufacturer’s instructions^3^.

### Statistical analyses

The data are presented as the mean ± standard deviation (SD). Two-tailed independent-sample *t*-test was used for comparisons between two groups. For comparisons of more than two groups, two-way ANOVA was performed (followed by Games-Howell test). Then Dunnett *t* test was used to explore differences between selected two groups. Rank data were analysed by the Wilcoxon signed rank test. All statistical comparisons were done using GraphPad Prism software version 6.0 (GraphPad Software, La Jolla, CA, USA) and SPSS 17.0 (SPSS, Chicago, IL, USA). *P* < 0.05 was regarded as statistically significant.

## Electronic supplementary material

Supplementary materials
