# Supplementary material for: Chronic vitamin D deficiency induces lung fibrosis through activation of the renin-angiotensin system
Source: Sci Rep. 2017 Jun 12;7:3312. doi: 10.1038/s41598-017-03474-6 (PMC5468249; doi:10.1038/s41598-017-03474-6)
Supplement: Supplementary file 1 — Supplementary materials [file 41598_2017_3474_MOESM1_ESM.pdf]

Chronic vitamin D deficiency induces lung fibrosis through activation of the renin-angiotensin system

Yongyan Shi[1], Tianjing Liu[2], Li Yao[1], Yujiao Xing[1], Xinyi Zhao[1], *Jianhua Fu[1],*Xindong Xue[1]

1. Department of Pediatrics, Shengjing Hospital of China Medical University, NO. 36 Sanhao Street, Shenyang, Liaoning 110004, P.R. China

2. Department of Pediatric Orthopedics, Shengjing Hospital of China Medical University, NO. 36 Sanhao Street, Shenyang, Liaoning 110004, P.R. China

S1

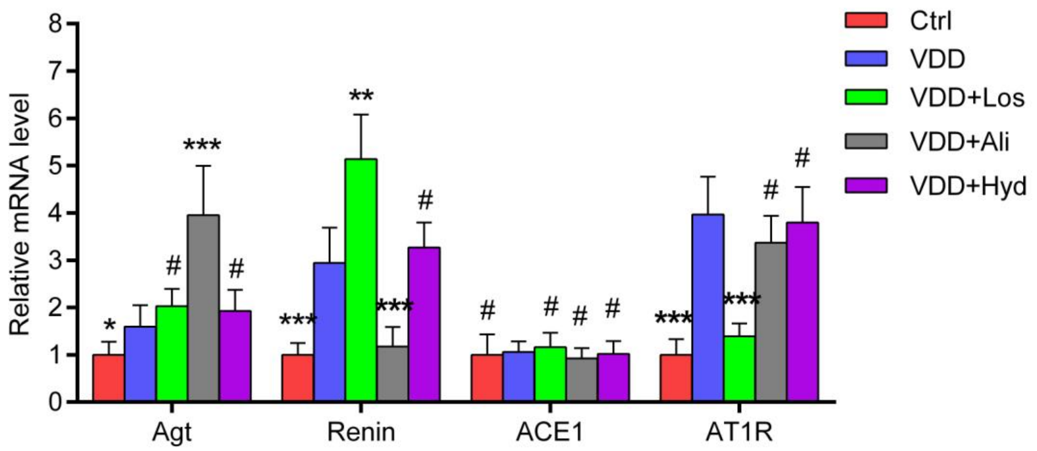

S1: Changes of relative mRNA expressions of RAS components after the administration of RAS blockers. #P >0.05, *P < 0.05, **P < 0.01, ***P < 0.001 compared to the VDD group (n=5-6 in each group). Ctrl: control; VDD: chronic vitamin D deficiency; VDD+ Los：chronic vitamin D deficiency treated with losartan; VDD+ Ali: chronic vitamin D deficiency treated with aliskiren; VDD+ Hyd: chronic vitamin D deficiency treated with hydralazine. Statistics: Two-way ANOVA and Dunnett *t*-test.

S2

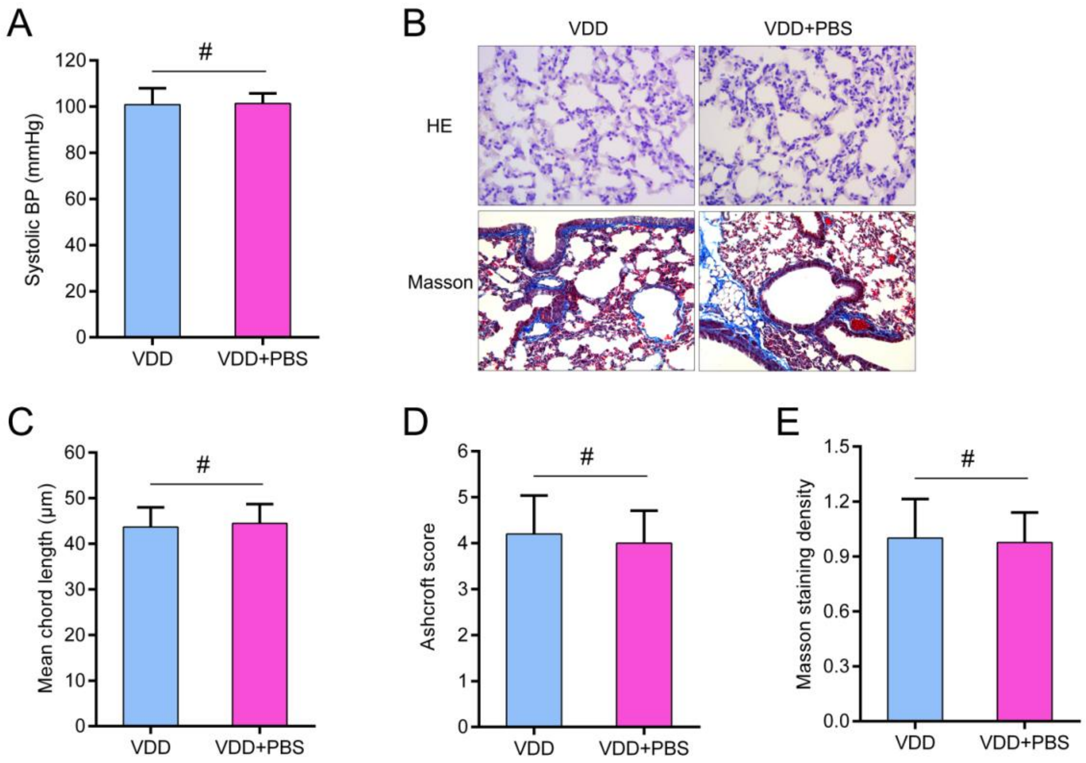

S2: Data of the control experiments for elimination of the influence of PBS. A. Blood pressure; B. Lung morphology; C: Chord length; D: Ashcoft scores E. Masson staining density. #: $P > 0.05$. n=6 in each group. Statistics: two-tailed independent-sample *t* test. VDD: chronic vitamin D deficiency; VDD+ PBS: chronic vitamin D deficient mice injected with the same volume of PBS as in the drug-treated groups.

S 3

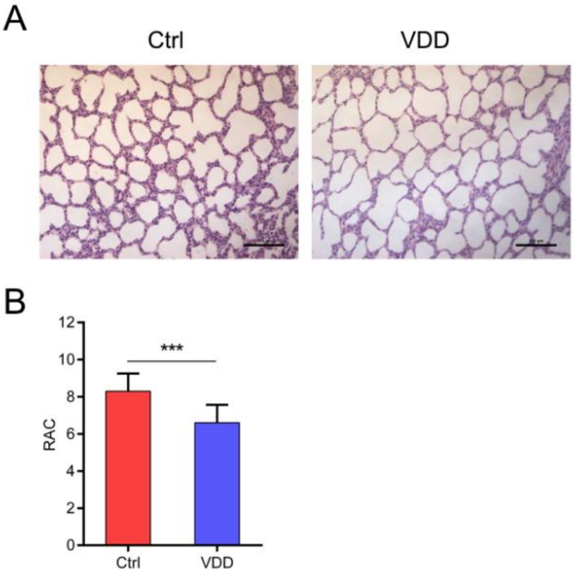

S3: Lung development on the day of birth. A. H&E staining of the lung tissue. Compared to the control group, alveolar simplification can be observed in the VDD group. B. Radial alveolar counts (RAC), an index to evaluate the stage of lung development, were significantly lower in the VDD group (6.60±0.31) compared to the normal group at P0 (8.30±0.30), $P < 0.001$, n=10. Ctrl: control; VDD: chronic vitamin D deficiency.

Fig 2

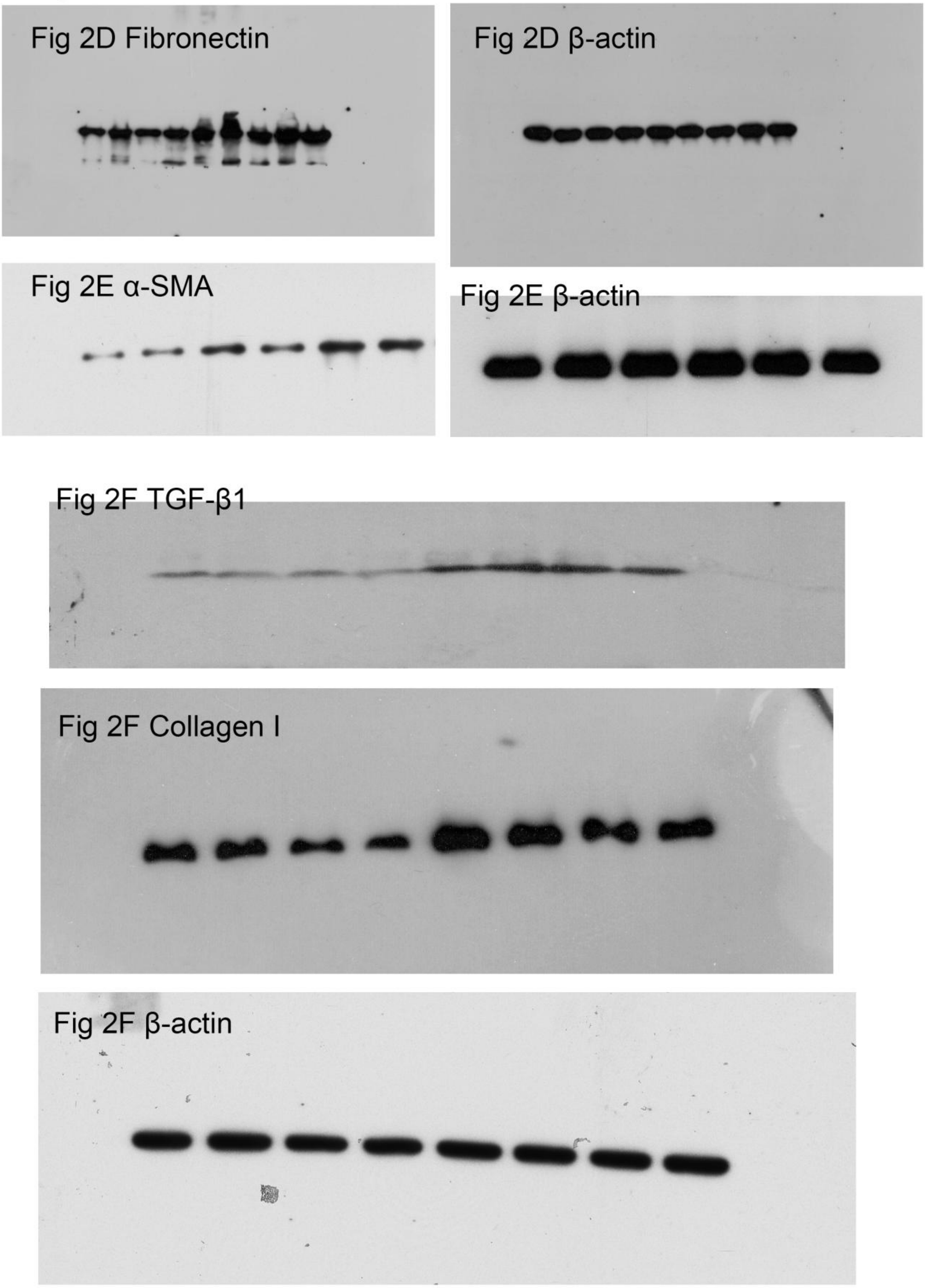
Fig 2D Fibronectin
Fig 2D β-actin
Fig 2E α-SMA
Fig 2E β-actin
Fig 2F TGF-β1
Fig 2F Collagen I
Fig 2F β-actin

Fig 5

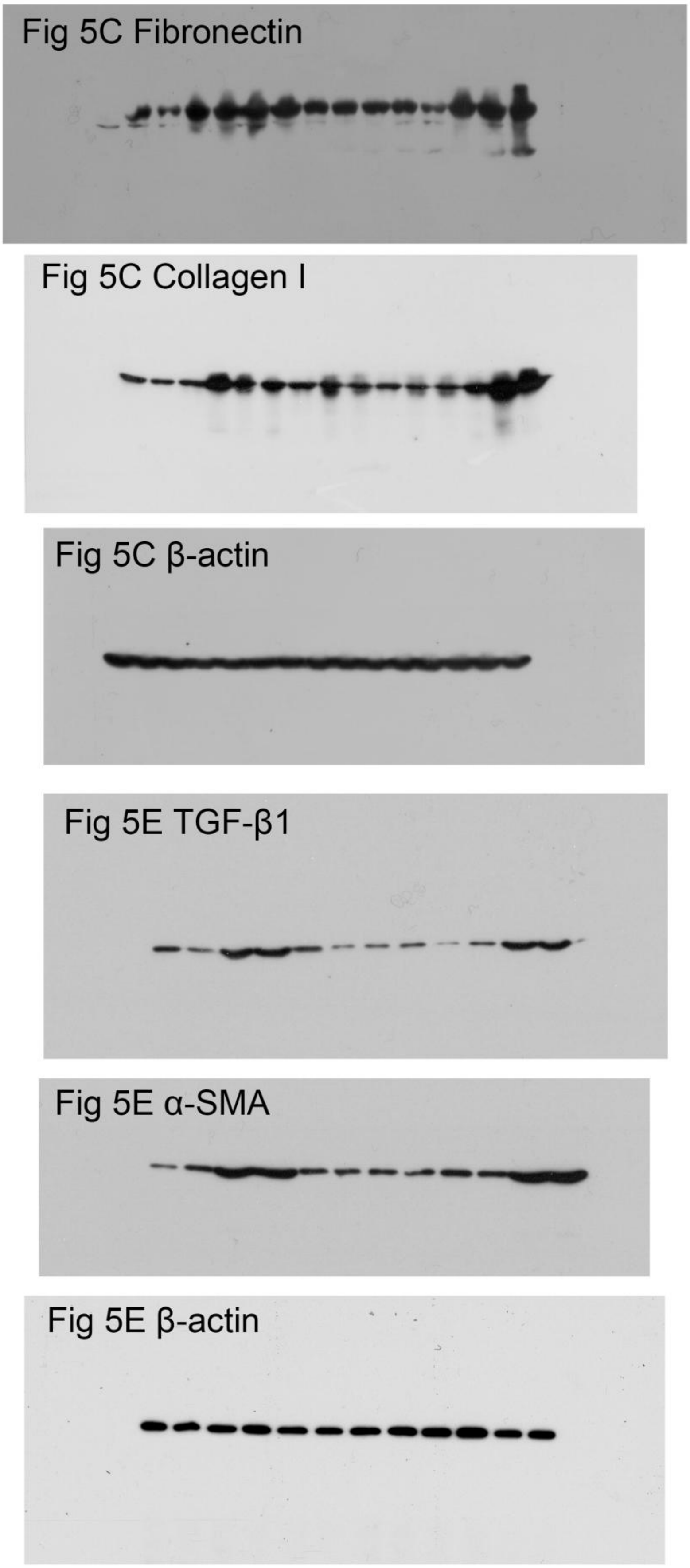
Fig 5C Fibronectin
Fig 5C Collagen I
Fig 5C β-actin
Fig 5E TGF-β1
Fig 5E α-SMA
Fig 5E β-actin
